# Assessment of clinical education (ASSCE): a scoping review of use and experiences of the tool

**DOI:** 10.1186/s12912-026-05379-0

**Published:** 2026-09-17

**Authors:** Anna Löfmark, Maria Engström, Gunilla Mårtensson

**Affiliations:** 1 Faculty of Health and Occupational Studies, University of Gävle, Gävle, Sweden; 2 Nursing Department, Medicine and Health College, Lishui University, Lishui, China

**Keywords:** Assessment tools, Clinical nursing education, Scoping review

## Abstract

**Background:**

Clinical practice in nursing education is perceived as important for students’ learning and professional development. One prerequisite for optimal learning is having assessment tools that support continuous development and assessment of students’ different abilities. The Assessment of Clinical Education (AssCE) tool has been used for over 20 years; however, less is known about how the tool is actually used and experienced both in clinical practice and in other areas of application.

**Aim:**

To describe and synthesize published literature on use and experiences of the AssCE tool.

**Methods:**

A scoping review using the framework of Arksey and O’Malley. The framework includes five stages: clarifying and linking the purpose and research question (Stage 1); identifying relevant literature (Stage 2); selecting literature (Stage 3) and extracting data (Stage 4); collating, summarizing and reporting results (Stage 5).

**Results:**

A total of 412 publications were found; 23 met the inclusion criteria and were included. The results show that the AssCE tool was used for various objectives mediating, above all, formative and summative feedback to students in a systematic and structured manner. The tool was utilized for planning the period, self-assessment, daily follow-up, and for assessment discussions with preceptors and the lecturer at the middle and end of a clinical period. Experiences of using the tool were mainly positive. Students and preceptors appreciated the tool as a structure for the clinical period content. Lecturers’ experiences of using the tool, although it was time-consuming, were that having well-prepared preceptors and students enabled deeper-level discussions. Lecturers supported developing the AssCE towards digitalization. Comparisons indicated benefits of the AssCE compared to other equivalent locally developed tools for clinical education. Research studies, alongside clinical education, showed that the AssCE tool was useful in other areas as well, for example, when nurses estimated their own competence.

**Conclusion:**

Students, preceptors, and lecturers found advantages of using the tool, as it was designed based on experiences of opportunities for dialogue and verification of students’ level of knowledge. The tool has also been used in new areas, indicating a need for further development and psychometric tests.

**Clinical trial number:**

Not applicable.

**Supplementary Information:**

The online version contains supplementary material available at https://doi.org/10.1186/s12912-026-05379-0.

## Background

In clinical nursing education, assessment tools are of crucial importance and intended to be used to promote a systematic, continuous assessment dialogue between the student, preceptor and lecturer about a student’s individual development in relation to the required learning outcomes [1, 2]. These tools are generally developed in accordance with guidelines determined by national boards of nursing or higher education requirements [3, 4]. Various assessment tools are in use internationally, but no standard exists [4–6]. Tools need continuous critical evaluation, as there are ongoing pedagogical changes and tendencies, for instance, within clinical learning environments and in relation to international guidelines for nursing education [6]. In Sweden, the Assessment of Clinical Education (AssCE) tool has been used for about two decades in nursing education. The present study aims to describe and synthesize published literature on use and experiences of the AssCE tool in the field of nursing by identifying all relevant literature regardless of study design, analyse the outcomes, identify knowledge gaps, draw conclusions from existing literature and guide further research.

A change in views on assessment in education has occurred. The focus has shifted from assessment of learning to assessment for learning [7, p. 478]. Assessment for learning refers to a concentration on the teaching and learning process to enhance learning to optimize all students’ ability and improve their achievements [8]. Assessment for learning is an interactive and dialogic pedagogical process involving understanding, progress and feedback. In the assessment process, students, preceptors and lecturers engage in discussion, explain their own thinking and experiences, share interpretations, and clarify their expectations with the purpose of promoting students’ learning [9]. Clinical practice offers continuous learning, and assessment should therefore provide support in the learning process. Here, influencing factors such as feedback, self-assessment and opportunities for reflection between the student, preceptor and lecturer are of the utmost importance [10].

Moreover, students can be a learning resource for one another [9, 11]. Assessment for learning focuses on the formative potential of the assessment through individualized feedback, where improvements have been noted and discussed. This underlines the notion that assessment can facilitate learning [12]. Students’ self-assessment has been seen as an important part of the assessment process –– a critical self-regulatory skill that students need [13]. Self-regulated learning, in turn, has been found to be related to nursing students’ metacognitive ability, self-efficacy and academic performance [14].

### The AssCE tool

The AssCE tool in focus was developed in the late 1990s in accordance with assessment for learning in clinical practice education and has been used for formative and summative assessment to enhance students’ learning and determine the status of learning achievement [8]. The tool was based on Swedish Higher Education qualification descriptors [15, 16] and international guidelines for nursing education [17, 18]. The tool has subsequently been revised with the support of evaluation studies and validated at the bachelor’s level [17, 18] using the Delphi method in groups of clinical nurse lecturers [3]. The tool has been used at the bachelor’s level in Sweden for about 20 years [19] and is currently used in more than half of Swedish universities [20]. AssCE at the master’s level was introduced in 2010 in Sweden and validated in a consensus group study in 2020. University lecturers were invited to consensus-building workshops as clinical experts given their overall responsibility for assessment meeting outcomes during students’ clinical practice [21]. In Norway, AssCE has been used at the bachelor’s and master’s level [22]. The tool comprises 21 factors divided into five areas: communication and teaching, the nursing process, examination and treatment, management and co-operation, and professional approach. A scale is linked to each factor and describes scoring criteria levels [3].

Each period of clinical practice education has specified learning outcomes stating what the student is supposed to know after the course. The AssCE tool provides guidance on how intended learning outcomes can be achieved. The assessment dialogue presupposes that students, preceptors, and lecturers collaborate and are jointly responsible for promoting and verifying students’ learning [23]. These 3-party meetings mean that different perspectives and knowledge can be brought together [1, 24]. The AssCE tool is used in these meetings to provide a regular and structured assessment dialogue. Although the tool has been used for over 20 years, and has been revised and evaluated, less is known about how the tool is actually used and experienced both in clinical practice and in other areas of application.

## Methods

A scoping review is considered suitable, as the study aim is to describe and synthesize areas of research by identifying all relevant literature regardless of study design, analyse the outcomes, identify knowledge gaps, draw conclusions from existing literature and guide further research [25, 26]. One strength of a scoping review is that it can address broader research questions and include a variety of study designs and methodologies, published and unpublished literature [27].

One formal approach to conducting a scoping review for broad research questions is provided by the framework originally designed in line with Arksey and O’Malley’s [25] methodological framework for scoping studies. The framework comprises five stages: Stage 1: identifying the research question; Stage 2: identifying relevant literature; Stage 3: selecting literature; Stage 4: charting data, and Stage 5: collating, summarizing, and reporting results [27]. The present scoping review will address a broad topic: how an assessment tool developed for the clinical part of nursing education has been used and discussed in the literature. Reporting of the present study has been guided by the Preferred Reporting Items for Systematic Reviews and Meta-Analysis Extension for Scoping Reviews, PRISMA-ScR [28].

### Identifying the research questions

The research questions were:

- How is the AssCE tool used by students, preceptors and lecturers in the assessment of nursing students during clinical practice in nursing education?
- What experiences do students, preceptors and lecturers have of using the AssCE tool during.

clinical practice in nursing education?

c. What are the results of studies in which the AssCE tool is examined in relation to other tools/instruments and used in contiguous areas of application?
d. What psychometric properties, validity and reliability are reported in relation to the AssCE tool?

### Identifying relevant studies

A search strategy was developed with assistance from an experienced librarian. The librarian proceeded from eight key articles suggested by the research team (marked * in the reference list). Key articles correspond to the research questions as closely as possible, are used as a starting point to identify further relevant articles and can be used as guidance for constructing the search strategy [29]. In Scopus and Web of Science, a search string was used to locate the eight key articles. The search string^1^ was adapted to each of the databases’ field tags. All eight articles were found in Web of Science. In Scopus, seven articles were indexed. After retrieving the key articles, the functions View cited by (Scopus) and Citation report (Web of Science) were used to download all citing articles. In Google scholar, the key articles were search individually and citations for each article were downloaded.

In April 2021, the main search was performed. At this time, the databases PubMed, SweMed+, SwePub along with the original databases Scopus, Web of Science and Google Scholar were used. In September 2021 and May 2024, a supplemental search was performed. To cover a subject in total, grey literature was searched in Google Scholar and Science Direct. In this case, the grey literature contained pedagogical reports, scientific reports and master’s theses. All studies aimed to describe, explore, or compare the subject under study [30].

### Study selection

All authors discussed and agreed on the inclusion and exclusion criteria. Selection of relevant studies was conducted using the PEO (Population, Exposure, Outcome) framework as well as the inclusion and exclusion criteria (Table 1). All study designs that used qualitative, quantitative, Delphi studies, systematic and integrative reviews were suitable for inclusion.

**Table 1:** Inclusion and exclusion criteria

	Inclusion	Exclusion
Population	Nursing students, preceptors, lecturers/teacher clinical experts, nursing researchers, nurses Context: clinical practice in nursing education (bachelor’s and master’s level), healthcare	Other healthcare students. Other types of healthcare staff Studies focusing solely on any other types of assessment of clinical education tool
**Exposure**	Use of the Assessment of Clinical Education (AssCE) tool	
**Outcome**	Experiences and use of the AssCE in clinical practice or in healthcare. Studies in which the AssCE is examined in relation to other tools/instruments or contiguous areas of application	

The main and supplemental searches included 412 (326, 16 and 70, respectively) publications. Duplicates were removed using Endnote according to the method presented by Bramer [31], leaving 254 studies.

The screening process was performed in two stages. First, titles and abstracts were screened independently by two authors (AL and GM), using the web-based tool Rayyan (rayyan.qcri.org), categorizing them as include, exclude or uncertain. If there were doubts, AL and GM discussed the study before a final decision was taken.

**Fig. 1:**
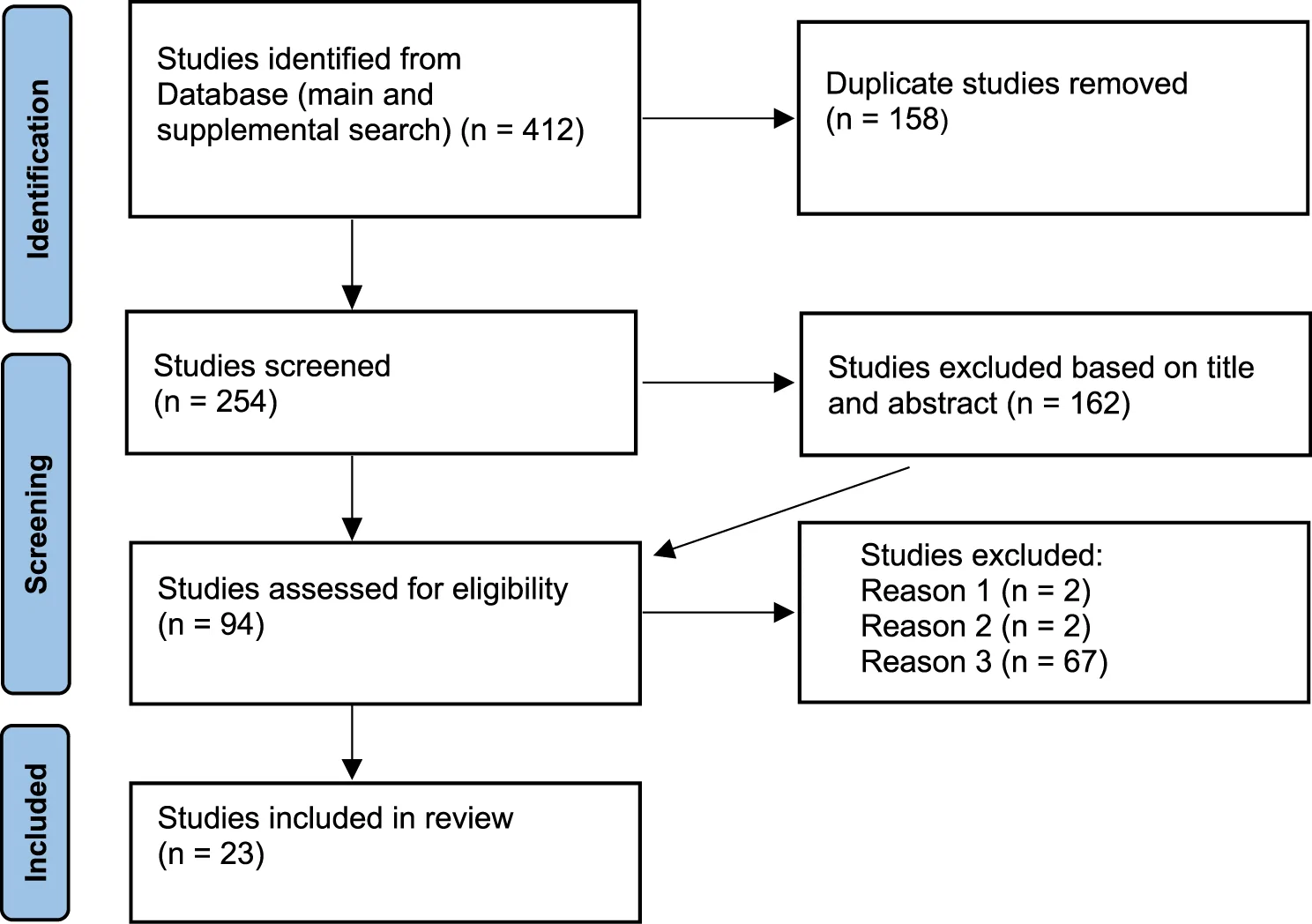
PRISMA flow chart showing study selection

In the second stage, the full texts of the eligible publications were reviewed. The 94 publications were screened in full text by one of the reviewers (AL). Any uncertainty concerning inclusion or exclusion was discussed by AL and GM. Of the 94 publications, 71 were excluded, leaving 23 eligible for summary. See Fig. 1 for reasons for exclusion. After consultation in the research group, 23 publications were included in the review.

### Charting data

The included studies were read by two authors (AL and GM). Data extraction was performed by AL in a charting document and checked for accuracy by the other researchers (GM and ME). Uncertainties and disagreements were resolved through discussion in the group. In the charting document, characteristics of each study were described to provide an overview. Summaries were developed for each study including author, country, sample, sample size, study design, data collection method, analysis technique, key findings and results (Appendix 1).

### Collating, summarizing, and reporting results

Summaries of the 23 selected studies were collated based on the research questions and presented in the results with subheadings: 1. Students’, preceptors’ and lecturers’ use of the AssCE tool; 2. Students’, preceptors’ and lecturers’ experience of using the AssCE tool; 3. Studies of the AssCE tool in relation to other tools/instruments; 4. Psychometric properties, validity, and reliability of the AssCE tool.

## Results

The included studies were conducted between 2004 and 2022 in Indonesia [1], Norway [7], Singapore [1] and Sweden [14]. Quantitative approaches were predominant (*n* = 10), followed by qualitative approaches (*n* = 7). Most were small-scale studies conducted at a single university and three Delphi studies, where most universities in Sweden and one university in Norway were represented. One systematic and one integrative review were included.

### Students’, preceptors’ and lecturers’ use of the AssCE tool

The studies show that AssCE was used in many different ways (Table 2). Objectives in studies of AssCE were mid-course discussions and final assessments, self-assessment, reflection, and discussions in conjunction with follow-up in clinical practice. Other examples were planning of supervision (preceptors), planning of the clinical practice period (students and preceptors) and planning of the 3-party discussions (lecturers) [1, 22, 32–35]. One master’s thesis described how the AssCE was used to form the basis and structure of the final assessment and the examination assignment [36].

**Table 2:** How the AssCE has been used

Publication	Country	^a^Field	^b^Level		^c^Objective					^d^User	^e^Preparation	^f^Original version Modified versions
			BNS Study Year	MNS Year	A = Mid- and final assessment	B=Self-assessment	C=Reflection/discussion	D=Planning	E=Clinical examination	1 = Students 2 = Preceptors 3 = Lecturers 4 = RN	1 = Yes 2 = No 3 = Unclear	O=Original M=Modified
Bengtsson and Wann-Hansson (2011)	Sweden	1	Year 2 and 3		x	x	x			1, 2, 3	3	O
Engström et al. (2017)	Norway	1	Year 3		x	x	x			1	1	O
Eulau et al., 2015	Sweden	1	Year 2		**x**	x	x			1	1	O
Islamoska (2014)	Sweden	3	Year 2 and 3		x	x	x			1, 2	3	O
Kauffeldt and Velander-Sundin (2009)	Sweden	1			x	x	x	x		2	3	O
Löfmark et al. (2016)	Norway	5		x	Final assessment	x	x	x		1, 2	1	O
Löfmark et al. (2019)	Norway	1	Year 3		x	x	x	x		3	1	O
Löfmark and Thorell-Ekstrand (2014)	Sweden	2 + 3	Year 1,2 and 3		x	x	x			1, 2	1	O
Vae et al. (2018)	Norway	1	Year 3		x	x	x			1, 2	1	O
Zetterlund (2019)	Sweden	1	Year 3 final week		Final assessment	x	x	x	x	2	1	O
Christiansen et al. (2020)	Norway	1	Year 1, 2 and 3	1–2	x	x	x			2	3	M
Josse-Eklund et al. (2014)	Sweden	4				x				4	3	M
Kydland and Nordström (2018)	Norway	2 + 3	Year 2		x	x	x	x		1, 2	1	M
Nordhagen et al. ( 2021)	Norway	1	Year 1–3		x	**x**	**x**			1, 2, 3	3	M
Lachmann and Nilsson (2021)	Sweden	1	Year 3 final week		Final assessment	x				1	3	
Nygårdh et al. (2017)	Sweden	1								3	3	
Putri and Sumartini (2021)	Indone-sia	2 and 3	x		x		x			1	3	
Ulfvarsson et al. (2018)	Sweden	6	x		x	x	x			1, 2	3	

^a^Field: 1 = general, different context; 2 = surgical; 3 = medicine; 4 = nursing homes, 5 = intensive and critical care, 6 = unclear

^**b**^ Level: BNS = Bachelor’s level nursing students; MNS = Master’s level nursing students

^c^Objective: A = Foundation for mid- and final assessment, final assessment or assessment seminars in groups B = Self-assessment of competency (student and preceptor) C= Reflection/discussion during assessment D = Planning for the clinical period, e.g., combined with work plans for the week E = structure for clinical examination

^**e**^Preparation: 1 = Yes, ordinary preparation, e.g., reading of information, preparation of cases, 2 = No, 3 = Unclear

^f^ O= Original version of AssCE, M = Modification of AssCE

Studies showed that the AssCE tool also has been used with a somewhat different design. The tool contained either more detailed learning outcomes for different levels and clinical periods or the same tool in a digitalized version [37, 38]. There was also a simplified version of the tool [39]. In one study, the AssCE was used by nurses working in community elderly care, where they self-rated their own nursing competence using the tool adapted for quantitative estimations [40].

Participants in studies about the use of AssCE were undergraduate students in different years of their programme (eight studies), master’s students (two studies), preceptors (seven studies) and lecturers (three studies). Sweden was represented in seven of the studies and Norway in seven.

### Students’, preceptors’ and lecturers’ experience of using the AssCE tool

It emerged in the reviewed studies that those who had used the tool had varied experiences. *Negative experiences*, according to some students, were that the AssCE statements were formulated in a general manner, making the tool difficult to apply in specialties such as psychiatry [32]. Students’ preparation prior to assessment discussions was associated with uncertainty and hesitation, and the preceptors’ feedback was sometimes more focused on what was left to learn than on individual students’ development and achievements to date [41]. Students’ *positive experiences* of using AssCE were that assessment discussions proceeding from the AssCE tool provided a clear structure and contributed to a supportive and encouraging discussion atmosphere; the tool was also considered beneficial in group assessment seminars [41, 42]. Using the tool became more self-evident after it was expanded by adding space for reflection and critical analysis of situations [43]. The language in the tool was experienced as understandable and useful [22]. The AssCE could be used for different years of the programme and could be combined with learning outcomes in various clinical courses [32].

Preceptors reported some n*egative experiences* as well. The generally formulated factors in the AssCE created difficulties in adapting the content to different specialities [32]. Furthermore, their own preparation prior to the assessment discussions created ambivalence and doubt [41]. Preceptors’ *positive experiences* highlighted the structured and systematic use of the tool in assessment discussions, in clinical examinations at the end of the education, and for planning supervision during the clinical period [33, 36, 41]. The atmosphere in the discussion meetings was mentioned as encouraging, and the assessment dialogue was facilitated by the fact that the language in the tool was easy to understand [22]. Students’ written reflections in the tool helped to deepen preceptors’ own knowledge [43]. Furthermore, the AssCE could be used throughout the programme and be combined with learning outcomes [32]. One study, a master’s thesis, described preceptors’ experiences of AssCE included in an examination document. They found that the document was easily understood and used [36].

Lecturers highlighted certain *negative experiences*; the AssCE was time consuming, and the concepts in the tool were sometimes vague and difficult to understand [1]. Lecturers reported *positive experiences* of, among other things, students’ and preceptors’ preparation and knowledge regarding the AssCE, which in turn affected the quality of the content and level of the discussion [1, 37]. Students’ and preceptors’ written reflections in the AssCE provided insights into students’ knowledge and development [39].

In some studies, consistent experiences emerged. In their *positive statements*, students and preceptors expressed appreciation for the clearly designed learning outcomes, with evident progression and a scale with levels to be used in the assessment process [32]. Students, preceptors, and lecturers felt the electronic version of the AssCE facilitated communication and gave new insights [39]. Other studies showed that there were opposing views in the group. The AssCE’s general and simplified formulations were perceived *positively* by some preceptors, who felt they facilitated adaptation to different clinical placements, while other preceptors pointed out the *negative* side, which was that the language was too theoretical, thus leading to alternative interpretations [38].

Participants in studies about experiences were undergraduate students in different years of their programme (12 studies) and master’s students (2 studies). Preceptors were represented in several studies [9], while lecturers were included in fewer [3]. Sweden was represented in ten of the studies and Norway in three. Supplement 1 provides additional information about students’, preceptors’, and lecturers’ negative and positive experiences.

### Studies of the AssCE tool in relation to other tools/instruments

The AssCE tool areas have been compared with the areas in the Nurse Professional Competence Scale – Short Form (NPC-SF), showing associations in assessment of nursing students’ professional and clinical competence ranging from *r* = 0.19 to 0.57, m = 0.42 [44]. AssCE has also been compared with the tool Assessment of Clinical Education (AClEd). The comparison favoured the AClEd tool, in some respects, for example it provided a clearer view of learning outcomes and enabled a fair and comprehensive assessment [45]. In another study, assessment tools used in nursing clinical education at all universities in Sweden were included in an investigation concerning the visibility of competencies for quality and safety standards, the so-called Quality and Safety Education for Nurses (QSEN) competencies. The AssCE tool was one of 23 tools examined, and these competencies were more visible in AssCE than in locally developed assessment tools [20]. In a randomized controlled trial, peer learning and problem-based learning (PBL) were introduced as pedagogical methods for nursing students, and scores from the AssCE tool were used as outcomes. Results showed that students in clinical learning using peer learning and PBL methods reported higher scores on various aspects of student competency achievement compared to a control group [46]. The AssCE tool has also been used in a study on healthcare for elders, a contiguous area alongside clinical education. The tool was transformed into a questionnaire, and nurses estimated their competence quantitatively in relation to the tool’s 21 factors [40].

### Psychometric properties, validity and reliability of the AssCE tool

The psychometric properties of the tool have been evaluated in various studies. See Table 3.

**Table 3:** Psychometric properties described for the AssCE tool

Publication	Validity				Differences between groups	Reliability		
	Face	Content	Construct	Criterion		Internal consistency	Test-retest	Inter-rater
Josse-Eklund et al. (2014)						0.78–0.85		
Lachmann and Nilsson (2021)	x					0.76–0.85, total scale 0.91		
Löfmark and Thorell-Ekstrand (2004)		x						
Löfmark and Mårtensson (2017)		x						
Mårtensson et al. (2020)	x	x						
Putri and Sumartini (2021)		x			x	Total scale 0.96		
Wu et al. (2015)	x	x		x				
Överbö et al. (2022)		x						

The AssCE tool for the bachelor’s level has been validated on two occasions using the Delphi technique to examine content validity [3, 47]. A Delphi study validated the tool for the master’s level, and both face and content validity were determined [21]. Lachmann and Nilsson [44] found associations ranging from *r* = 0.19 to 0.57 when investigating associations between the areas in the AssCE and the factors in the NPC. In the study, all areas were examined for associations, not only those with related content. The tool, when used as a research instrument, has been able to detect differences between groups, and reliability measured as internal consistency was reported at 0.96 for the whole instrument [41]. Josse-Eklund et al. [40] used the AssCE when nurses estimated their competence – on a 5-point Likert scale from 1 (very weakly developed ability) to 5 (very strongly developed ability) – in a study on individual and organizational factors that could potentially influence registered nurses’ attitudes towards patient advocacy. They reported Cronbach’s alpha values for the different areas, ranging from 0.78 to 0.85, and 0.94 for the total scale. Results of test-retest, interrater reliability and construct validity have not been reported in any of the included studies.

Two review studies were also included in the study selection. The AssCE tool was one of 14 other assessment tools in a systematic review by Wu et al. [4]. The AssCE was characterized as having been developed with reference to Swedish higher education, reported to have face and content validity in discussions with various nursing experts, and described as a criterion-referenced tool. The integrative review by Øvrebø et al. [48] evaluated postgraduate critical care nursing education methods and tools. The AssCE master tool was one of fifteen included tools. The AssCE was singled out for its information to students, preceptors, and lecturers, with objective examples and rating scales contributing to open discussions and reflections and supporting the learning process.

## Discussion

The aim of the present scoping review was to describe and synthesize published literature on use and experiences of the Assessment of Clinical Education (AssCE) tool in the field of nursing education and nursing. The results show that the AssCE tool was used for various objectives mediating, above all, formative and summative feedback to students in a systematic and structured manner. The tool was utilized for planning of the period, self-assessment, daily follow-up, and for assessment discussions with preceptors and a lecturer at the middle and end of a clinical period. Experiences of using the tool were mainly positive. Students and preceptors appreciated the tool as a structure for the clinical period content. Lecturers’ experience of using the tool, although it was time-consuming, was that having well-prepared preceptors and students enabled deeper-level discussions. Lecturers supported developing AssCE towards digitalization. Other findings that need to be highlighted and are crucial to generating new and in-dept knowledge in further development of the AssCE are studies in which the tool was compared to other assessment instruments; in one study, the tool was used as an instrument for nurses’ assessment of their own competence. Overall, we found important and unknown areas of use for the AssCE, which highlights the need to further develop and psychometrically test the tool.

Many aspects of the learning process need to be realized, and the present review shows that the AssCE tool provides opportunities for meeting different requirements. In most studies, the tool was used as intended, for formative and summative feedback during mid-course and final assessment at the undergraduate and postgraduate level of clinical education. Students, preceptors and lecturers have used the AssCE as a support for continuous learning and for different purposes: for planning clinical periods [33], as a structure for daily follow-up, for structure and systematic use in the mid-course discussions and final assessments, for self-assessment and reflection [32, 41, 43]. The AssCE constituted a joint tool in assessment discussions, created a framework for a supportive atmosphere, and enabled reflection and discussions between the participating students and preceptors [41, 42]. Using the tool for written reflections strengthened the learning process and gave opportunities for feedback [46]. In discussions of the AssCE’s content, the students were able to connect the content to the learning environment they were part of [38].

Experiences of using the AssCE tool in nursing and clinical practice can be summarized as predominantly positive, although there were also opinions about uncertainty in connection with one’s own preparation for the assessment discussion. Students explained that provision of feedback from preceptors and lecturers was facilitated by having a structured supportive conversation climate in the discussion [41], which is a prerequisite for experiencing empowerment, motivation, and self-efficacy. But there were also examples of the opposite, of a discussion climate that was uncomfortable and tense [41]. Nicol and MacFarlene-Dick [49] stressed that formative assessment and feedback should be used to strengthen students in their effort to take control over their own learning, and to promote independence by making students aware of their own thinking. As a self-regulated learner, it is also important to be aware of gaps in one’s own knowledge and skills and to know where to go next in the learning process [2]. Lectures emphasized that the discussion content was dependent on participants’ preparation, and discussions supported by the factors in the tool could increase the level of discussion and validate students’ learning [1, 37], which is crucial to learning [8].

The AssCE tool was developed for self-assessment; there is a scale for each factor with described criteria for different levels. It appears that students and preceptors felt the ratings they were required to make in their respective tools, and did in most cases, were a good starting point that facilitated dialogue and discussions on the development of students’ knowledge and achievements [32]. But self-assessment was also experienced as challenging when preparing for assessment meetings. Both students and preceptors expressed uncertainty and hesitation about estimating the abilities described in the tool [41]. Results from earlier studies have pointed to the importance of using understandable language in the learning outcomes, which is necessary for students to achieve an independent role in the assessment process [2], and the importance of self-assessment in encouraging personal variables such as self-efficacy [13, 50]. Having clear goals during clinical practice is essential to improving constructive feedback during clinical learning [10]. Goals are established as learning outcomes in the course syllabus and, together with the AssCE tool content, they constitute guidelines for what a learner should know or be able to demonstrate after completion of a course.

In many countries, continuous formative assessment is done by preceptors during the clinical practice education period, while clinical lecturers alone are responsible for the final grading. Grading is the conclusion of a decision-making process, often supplemented with a clinical examination [51]. According to Taras [52], assessment in education often appears to be more concerned with grades than with learning. In its current design, the AssCE is not meant to be used for grading performance, but as a basis for a dialogic pedagogical learning process. In the reviewed studies, it is emphasized that it is in this pedagogical learning process that the AssCE tool has its strength and value [1]. However, use of a tool like the AssCE together with clinical examinations can constitute one part of a final examination.

Two examples in the review concerned modification of the AssCE tool, one with more detailed learning outcomes and clear progression and another where the modified tool was developed into an electronic assessment version. The changes were evaluated positively. Students became more active in their self-assessment when this also offered opportunities for discussion about levels of knowledge [37, 41]. The benefit mentioned when this tool was computerized was that the continuity of the communication between students, preceptors and lecturers increased significantly [39]. New ways of using the AssCE tool were revealed in a study in which nurses self-assessed their abilities on all 21 factors related to nurses’ daily work [40]. This experience can pave the way for possible new areas of use, as well as use of the tool to generate outcomes in a randomized controlled trial [46].

Some students and preceptors considered the language in the AssCE to be difficult to apply in certain specialist clinics [32]. According to Immonen et al. [10], the language in assessment tools and assessment criteria must be objective and clear. Creative ability and dedication are required to adapt the AssCE’s general content for use in a variety of healthcare fields. Lecturers have the important task of providing support when these problems arise.

Comparisons with other similar instruments and tools are important to understanding the quality and usefulness of any given tool. Comparisons between the NPC-SF Scale and the AssCE showed relationships of moderate strength [45]. However, all areas were examined, and for some areas the associations are thought to be weaker. Comparisons with different tools used at universities in Sweden showed that QSEN competencies were more visible in AssCE results than in results from locally developed assessment tools [20]. The ACIEd, a similar tool, used for the same purpose as the AssCE, showed differences in, for example, users’ understanding of learning outcomes and possibilities for fair assessment. Continued development of tools is of great importance to being able to compare, and thus gain more knowledge about, learning during clinical practice education. Also crucial is having validated assessment tools and a critical analysis of the assessment discussions, which still constitute a somewhat unexplored area of research [53]. If the AssCE is to be used as a research instrument, there is a need for further validation and tests of reliability in different contexts.

### Methodological considerations

The present review is consistent with descriptions of scoping reviews, in that breadth is sought at the expense of depth. It contains both scientific and grey literature. The search strategy was comprehensive, and a wide range of databases was used. However, there is no guarantee that all studies relevant to the scoping review have been identified. Inclusion and exclusion criteria were firmly set early in the process, and we strictly adhered to them throughout the data collection process. The strict procedure used minimized the possible risk of bias. Stage 1 and 2 (identifying the research question and relevant studies) were performed by two researchers, and the other stages were performed by all the researchers. One aspect we have been very aware of is that the literature reviewed includes studies in which we have been involved as researchers, a limitation we are very much conscious of and have tried to handle with the greatest discretion. Those studies have undergone peer review before being accepted by the scientific journals, and extraction of data for the review has strictly followed the guidelines for scoping reviews.

In recent years, a new application has been developed: Technology, Optimized Practice Process in Nursing (TOPP-N). In this application, the AssCE tool is one of several elements [54]. In the published study, no findings specifically linked to the AssCE tool can be found, and this study was therefore excluded from the review.

Continuous use by students, preceptors and lecturers during clinical practice and in the 3-year nursing programme at many universities in Sweden, for about the past 20 years, means that the tool is well-established. Many preceptors have personal experiences of the tool from their own nursing education. To our knowledge, no other assessment tool has such a long history. During this period, the AssCE has undergone development –– for example, addition of clearer formulations of the factors, space for reflections and electronic versions –– which has facilitated communication between students, preceptors and lecturers. Recurrent evaluations, adaptation to changing international guidelines and pedagogical influences have also influenced development of the tool.

## Conclusion and implication for future research

The strength of the AssCE is that the tool is validated and used at both the undergraduate and postgraduate level and can be combined with learning outcomes in relation to the respective courses. The tool has also been used for self-assessment, a crucial part of the learning process, and something that contributes to empowering students to become self-regulated learners. The present review revealed that students, preceptors and lecturers have found many advantages in using the AssCE as support for a continuous learning process, implying constructive feedback and reflections between the student, preceptor and lecturer in an interactive and dialogic pedagogical process. Perceived difficulties in using AssCE were that the language in the tool was considered somewhat theoretical and that the generally formulated factors created difficulties in adapting the tool to different specialties. Studies of comparable assessment instruments are few, but continued development of assessment instruments is important. Assessment of students’ knowledge and achievements during clinical practice is decisive in ensuring the quality of nursing education and nursing.

The present review shows that the AssCE has also been used in ways different from what it was developed for, indicating that there remain paths for further use and development. If the tool is to be used to evaluate interventions, it needs to be tested for, e.g., construct validity and test-retest reliability, even though the results thus far regarding, for example, differences between groups and internal consistency are promising. Other questions for future research are: In what way has the AssCE, as a tool, been of crucial importance in determining pass or fail when evaluating students’ clinical practice education? Are there factors in the tool that are more crucial in this regard?

## Supplementary Information

Below is the link to the electronic supplementary material.

Supplementary Material 1

Supplementary Material 2

## Data Availability

No datasets were generated or analysed during the current study.

## Footnotes

1 “https://doi.org/10.1111/j.1471-6712.2000.tb00568.x OR “https://doi.org/10.1111/j.1365-2648.2004.03198.x OR “https://doi.org/10.1016/j.nepr.2013.08.015 OR “https://doi.org/10.18261/issn.1892-2686-2016-04-05 OR “https://doi.org/10.1016/j.nedt.2017.01.009 OR “https://doi.org/10.1016/j.nedt.2016.12.009 OR “https://doi.org/10.1016/j.nepr.2017.11.014 OR “https://doi.org/10.1016/j.nepr.2019.02.010 OR “https://doi.org/10.1016/j.nepr.2020.102741.
