# Supplementary material for: Assessment of clinical education (ASSCE): a scoping review of use and experiences of the tool
Source: BMC Nurs. 2026 Sep 17;25:862. doi: 10.1186/s12912-026-05379-0 (PMC13587395; doi:10.1186/s12912-026-05379-0)
Supplement: Supplementary file 1 [file 12912_2026_5379_MOESM1_ESM.docx]

**Supplement 1. Students’, preceptors’ and lecturers’ experiences of using the AssCE**

| **Publication** | **Positive** |  |  | **Negative** |  |  |
| --- | --- | --- | --- | --- | --- | --- |
|  | **Students** | **Preceptors** | **Lecturers** | **Students** | **Preceptors** | **Lecturers** |
| Bengtsson and Wann-Hanssson (2011) | AssCE became clearer when the tool expanded with space for reflections that involved considerations and feedback. | Students´ written reflections in the AssCE helped to deepen knowledge about episodes in daily clinical work. | Students´ and preceptors´ written reflections in the AssCE provided information about students´ knowledge and development. |  |  |  |
| Engström et al. (2017) | Perceptions of using the AssCE and of the clinical learning environment were linked to the content discussed during assessment discussions. |  |  |  |  |  |
| Eulau et al. (2015) | Assessment based on the AssCE in student- preceptor dialogues throughout the clinical practice period combined with assessment seminars in groups at the end of the period was successful. It helped facillitate a deep approach to learning. |  |  |  |  |  |
| Kauffeldt and Velander-Sundin (2009) |  | The AssCE was used to give structure to the assessment discussions, for reflective development dialogue and for planning supervision. |  |  |  |  |
| Löfmark and Thorell- Ekstrand (2014) | The tool can be used during different years of the programme and can be combined with learning outcomes. Using the scale facilitated the assessment dialogue and illustrated development. Students felt they were almost always prepared with self-assessment. | The tool can be used during different years of the programme and can be combined with learning outcomes. Using the scale facilitated the assessment dialogue and illustrated development. Having formulated overall learning outcomes for the three years as useful in the assessment process. Students were prepared with self-assessment. |  | Factors in the AssCE are too general. Difficulties in adapting the content to different clinical placements. | Factors in the AssCE ar too general. Difficulties in adapting the content to different clinical placements. |  |
| Löfmark et al. (2016 ) | The AssCE was found to be understandable and useful for dialogue during the assessment of students in intensive and critical care education. | The AssCE was found to be understandable and useful for dialogue in assessment of students in intensive and critical care education. |  |  |  |  |
| Löfmark et al. (2019) |  |  | Content of assessment discussions depended on participants’ preparation. Discussion of factors could verify students learning. The AssCE could raise discussions to a higher level. |  |  | The AssCE is time-consuming. Vague concepts. |
| Vae et al. (2018) | Systematic and structured use of all the factors in the tool. Situations from clinical practice were highlighted.  Examples of supportive, calm and encouraging atmosphere in the assessment discussions. | Systematic and structured use of the tool and factors were illustrated with concrete examples. Supportive atmosphere in the assessment discussions. |  | Preparation for assessment discussions entailed uncertainty and hesitation. Feedback concentrated more on learning to come than on individual students' development and achievements. Examples of discussion climate as uncomfortable, tense. | Preparation for assessment discussions entailed uncertainty and hesitation. |  |
| Zetterlund (2019) |  | Examination document was easy to use, had a clear structure.  Examination of students was also a personal process. |  |  | Experiences of inadequacy and uncertainty. Time-consuming. |  |
| Christiansen et al. (2020) |  | General formulations in the tool gave opportunities to adapt the learning outcomes to the particular clinical placement. |  |  | Language used in the assessment tool unclear and theoretical, also difficult to use. Preceptors experienced their role in the assessment discussions as passive. |  |
| Kydland and Nordström (2018) | The modified AssCE was helpful, with an emphasis on progression, learning outcomes and criteria for establishing the level of competence.  Using the scale in the tool enabled a discussion about levels of knowledge and skills. | The modified AssCE was helpful, with an emphasis on progression, learning outcomes and criteria for establishing the level of competence.  Using the scale in the tool enabled a discussion about levels of knowledge and skills. |  |  |  |  |
| Nordhagen et al. (2021) | Students received feedback more often and were more active in documenting reflections. | Preceptors were more prepared for assessment of students. | Lecturers could take part in the written communication between student and preceptor. |  |  |  |
