# Supplementary material for: Assessment of clinical education (ASSCE): a scoping review of use and experiences of the tool
Source: BMC Nurs. 2026 Sep 17;25:862. doi: 10.1186/s12912-026-05379-0 (PMC13587395; doi:10.1186/s12912-026-05379-0)
Supplement: Supplementary file 2 [file 12912_2026_5379_MOESM2_ESM.docx]

**Appendix 1. Characteristics of included studies**

| **Author, year, peer reviewed** | **Country** | **Sample** | **Sample size** | **Design** | **Data collection method** | **Analysis technique** | **Key findings and results** |
| --- | --- | --- | --- | --- | --- | --- | --- |
| Bengtsson and Wann-Hansson (2011)  Scientific report | Sweden | Students, preceptors lecturers | 7  3  6 | Descriptive | Focus group interviews | Qualitative content analysis  No final evaluation | * The AssCE was included in a portfolio. The five areas in the AssCE were added with a space where students were supposed to write their own reflections.  * Students, preceptors and lecturers had different experiences of the written reflections in the AssCE – consideration (students), deepens knowledge (preceptors), an important part of the learning process (lecturers). |
| Engström et al. (2017)  Peer reviewed | Sweden | Students | 110 | Cross-sectional  Correlational | Questionnaire | Logistic regression analysis | * The AssCE supported students' clinical learning, and this link, in turn, was mediated  by the content discussed during their mid-course discussion and final assessment.  * The AssCE contributed to assessment discussion about students´ knowledge, skills, and professional judgement and, in turn, to clinical learning. |
| Eulau et al. (2015)  Peer reviewed | Sweden | Students | 20 | Descriptive | Qualitative survey | Qualitative content analysis | * The AssCE was used as a structure for case seminars in groups together with a preceptor in the middle of the period.  * Active participation in the summative assessment discussions with a teacher can mean a deeper approach to learning and understanding. |
| Islamoska (2014)  Pedagogical report | Sweden |  |  |  |  | No evaluation | * Factors in the AssCE were extended additionally with text about the daily care nurses give.  * Added to the examples were examples of skills related to Good achievement of goals, Very good achievement of goals and Inadequate achievement of goals. |
| Kauffeldt and Velander-Sundin (2009)  Scientific report | Sweden | Preceptors | 199 | Descriptive | Questionnaire | Frequencies | * The AssCE was used as a foundation for the structure of the assessment discussions and reflective development dialogue.  * The AssCE was used as a structure for planning supervision. |
| Löfmark et al. (2016)  Peer reviewed | Norway | Students  Preceptors | 7  7 | Descriptive | Group interviews | Qualitative content analysis | *Translation process in five steps with forward translation, back translation and comparison.  *Limited testing of the tool with students and preceptors showed that the AssCE was understandable and useful for dialogue during the assessment of intensive and critical care nursing students. |
| Löfmark et al. (2019)  Peer reviewed | Norway | Lecturers | 14 | Descriptive | Repeated interviews | Qualitative content analysis | * Content of assessment discussions depended on participants’ preparation  * Discussions in the 3-party meetings verified whether concepts were understood.  * Discussing examples of factors could verify students’ learning.  * The AssCE could raise the discussions to a higher level.  * The AssCE provided opportunities to include explanations as support. |
| Löfmark and Thorell-Ekstrand (2014)  Peer reviewed | Sweden | Students Preceptors | 191  101 | Descriptive | Questionnaire | Frequencies and summary statistics | * The revised AssCE could be used during different years of the programme and factors in the AssCE form could be combined with learning outcomes in the course syllabus. * The scale added to each factor facilitated the assessment dialogue and offered possibilities to illustrate the students’ development during clinical periods.  *Formulated overall learning outcomes on the front page of the tool showed the demands of progression during the three years of clinical education and were useful in the assessment process. |
| Vae et al. (2018)    Peer reviewed | Norway | Dyads of students- preceptors | 13 | Descriptive | Interviews | Qualitative content analysis | * The AssCE was used in a very systematic and structured way.  * Preparation for assessment discussion caused uncertainty and hesitation.  * Feedback emphasized assignments in the next part of the education, but less about the individual students' progress. |
| Zetterlund (2019)  Master´s thesis | Sweden | Preceptors | 7 | Descriptive | Interviews | Thematic analysis | *Examination document composed of the AssCE and NKSE (National Clinical Final Examination) called KSE.  *The examination document was easy to use, had a clear structure.  *Students´ reflections in their diaries were used in the discussion topics during the examination. |
| Christiansen et al. (2020)    Peer reviewed  Modified | Norway | Preceptors/mentors | 33 | Descriptive | Interviews | Qualitative content analysis | *A modified AssCE, where learning outcomes were found to be too general and theoretical, leading to different interpretations and resulting in divergent views on students´ competence.  *Some mentors appreciated the general formulations in the tool  *Mentors experienced their role in the assessment discussions as passive. |
| Josse-Eklund et al. (2014)  Peer reviewed  Modified | Sweden | Nurses in healthcare for elders | 226 | Descriptive | Questionnaire | Multiple stepwise regression analysis | *A modified version of the AssCE was used for self-assessment of nursing competence.  *Two factors in the AssCE – Performing the nursing care process and Supervision of work and cooperation – influenced nurses’ attitudes towards patient advocacy. |
| Kydland and Nordström  (2018)  Peer reviewed  Modified | Norway | Students  Preceptors | 129  18 | Descriptive | Questionnaire | Frequencies | *This modified AssCE had different content for the various periods of clinical education. Students and preceptors found that making learning outcomes more specific was helpful.  *The assessment tool can be used at various clinical education levels, and the scale makes it possible to show the student’s progress.  *Students had prepared themselves prior to assessment and participated actively in the assessment discussions, which may promote learning. |
| Nordhagen et al. (2021)  Peer reviewed  Modified | Norway | Students , Preceptors Lecturers | 139  180  25 | Descriptive | Questionnaire | Frequencies | * The AssCE was modified to an electronic assessment document, which facilitated the student assessment situation.  * Preceptors were more prepared, and their view was more in accordance with lecturers’ concerning assessment.  * Students received feedback more often and were more active in documenting their reflections.  * Lecturers could follow the written communication between student and preceptor, which gave greater knowledge and insight. |
| Lachmann and Nilsson (2021)  Peer reviewed  Another area of use | Sweden | Students | 151 | Cross-sectional Correlational | Questionnaire | Bivariate correlations analysis | * Statistically significant correlations between the NPC-SF Scale and the AssCE tool.  * The NPC-SF and the AssCE could be used as tools for nursing students to reflect on their professional theoretical and clinical competence and how it has developed during their studies. |
| Nygårdh et al. (2017)  Peer reviewed  Another area of use | Sweden | Assessment tools | 23 | Descriptive | Audit template | Descriptive statistics and comparative analysis | * Investigation of the six QSEN competencies, where AssCE was one, showed that teamwork and collaboration was the most visible competency.  * Patient-centered care was visible to a large degree and informatics was the least visible.  * The key characteristics were significantly more visible in the AssCE assessment tool than in the locally developed assessment tools. |
| Putri and Sumartini (2021)  Peer reviewed    Another area of use | Indonesia | Students, preceptors | Treatement group 16, control group 15 | Experimental  Randomized controlled trial (RCT) | Questionnaire | Bivariate (independent t-test) | *The AssCE was the dependent variable, where the intervention was integrated peer- learning and problem-based learning methods.  * Peer learning and PBL can affect clinical learning outcomes with respect to communication and teaching, nursing care, examination and treatment, and professional approaches. |
| Ulfvarson et al. (2018)  Peer reviewed  Another area of use | Sweden | Students, Preceptors | 435  54 | Cross-sectional, Comparative | Questionnaire | Cross-tabulation  Chi-square tests | *The criterion-referenced assessment instrument AClEd was perceived both by nursing students and clinical supervisors to promote greater understanding of the learning outcomes and the possibility of a fair, unbiased, and understandable assessment. |
| Löfmark and Thorell-Ekstrand (2004)  Peer reviewed  Development and validation | Sweden | Lecturers / researchers | 25 in round 1 and 21 in round 2 | Descriptive | Delphi rounds | Delphi technique | *Two new factors Collaboration with family and society and Independence.  * Two altered formulations of areas - Professional stance and The nursing process.  *Altered formulation of the factor Scientific awareness in the area Professional stance |
| Löfmark and Mårtensson (2017)    Peer reviewed  Development and validation | Sweden | Clinical nurse lecturers | Stage 1: 35 in three Delhi rounds. Stage 2: 46 lectures | Descriptive | Delphi rounds Questionnaire | Delphi technique  Descriptive statistics | *Written communication in Delphi rounds concerning formulation of factors in the AssCE and suggested changes until consensus was reached.  *Validation of the revised AssCE was established with high congruence between the factors in the AssCE and examined learning outcomes. |
| Mårtensson et al. (2020)  Peer reviewed  Development and validation | Sweden | Lecturers in specialist and midwifery education | 28–56 participants at each of the five workshops | Descriptive | Group discussions in workshops, Questionnaire | Delphi technique  Descriptive statistics | * Clinical experts view the tool as valid and verified in the audit and found that learning outcomes in different clinical courses corresponded well with the content of the revised AssCE-master tool.  * The AssCE-master tool is used for providing structure and content in assessment discussions, including feedback, support and appraisal of learning progress as the most important elements.  * The AssCE provided clarity and structure, making it easier to discuss concrete goals. |
| Wu et al. (2015)    Peer reviewed | Singapore |  | 14 studies | Systematic review |  |  | *The AssCE was characterized as having been developed with reference to Swedish higher education  * The AssCE has face and content validity and is a criterion-referenced tool.  * A holistic model of clinical assessment tool |
| Överbö et al. (2022)    Peer reviewed | Norway |  | 15 studies | Integrative review |  |  | * The AssCE is a valid assessment tool with objective examples, rating scales with marked intervals contribution to clarity and feedback to students, preceptors and lectures.  * The AssCE has content validity |
